# Reference curves of birth weight, length, and head circumference for gestational ages in Yogyakarta, Indonesia

**DOI:** 10.1186/s12887-016-0728-1

**Published:** 2016-11-21

**Authors:** Ekawaty L. Haksari, Harrie N. Lafeber, Mohammad Hakimi, Endy P. Pawirohartono, Lennarth Nyström

**Affiliations:** 1 Department of Child Health, Faculty of Medicine, Gadjah Mada University, Sardjito General Hospital, Jl. Kesehatan No. 1, Yogyakarta, 55284 Indonesia; 2 Department of Pediatrics, Vrije Universiteit Medical Center, P.O. Box 7057, 1007 MB Amsterdam, The Netherlands; 3 Faculty of Medicine, Gadjah Mada University/Sardjito General Hospital, Jl. Kesehatan No. 1, Yogyakarta, 55284 Indonesia; 4 Public Health and Clinical Medicine, Umeå University, SE-901 87 Umeå, Sweden

**Keywords:** Reference curve, Birth weight, Supine length, Head circumference, Sex, First-later-born children, Preterm term

## Abstract

**Background:**

The birth weight reference curve to estimate the newborns at risk in need of assessment and monitoring has been established. The previous reference curves from Indonesia, approximately 8 years ago, were based on the data collected from teaching hospitals only with limited gestational ages. The aims of the study were to update the reference curves for birth weight, supine length and head circumference for Indonesia, and to compare birth weight curves of boys and girls, first child and later children, and the ones in the previous studies.

**Methods:**

Data were extracted from the Maternal-Perinatal database between 1998–2007. Only live singletons with recorded gestational ages of 26 to 42 weeks and the exact time of admission to the neonatal facilities delivered or referred within 24 h of age to Sardjito Hospital, five district hospitals and five health centers in Yogyakarta Special Territory were included. Newborns with severely ill conditions, congenital anomaly and chromosomal abnormality were excluded. Smoothening of the curves was accomplished using a third-order polynomial equation.

**Results:**

Our study included 54,599 singleton live births. Growth curves were constructed for boys (53.3%) and girls (46.7%) for birth weight, supine length, and head circumference. At term, mean birth weight for each gestational age of boys was significantly higher than that of girls. While mean birth weight for each gestational age of first-born-children, on the other hand was significantly lower than that of later-born-children. The mean birth weight was lower than that of Lubchenco’s study. Compared with the previous Indonesian study by Alisyahbana, no differences were observed for the aterm infants, but lower mean birth weight was observed in preterm infants.

**Conclusions:**

Updated neonatal reference curves for birth weight, supine length and head circumference are important to classify high risk newborns in specific area and to identify newborns requiring attention.

## Background

Size at birth reflects fetal growth and health as well as provides important information on the newborns infant. Many studies have been carried out to construct a theoretical birth weight curve for gestational age [1, 2]. The birth size curve was used as a reference to facilitate prediction of growth, estimate the risk for small gestational age (SGA), and to identify newborns at risk that require assessment and monitoring during the neonatal period [3–7].

The prevalence of high risk newborns depends on the birth curve used [8]. Therefore, a perinatal growth chart that is versatile enough to serve as an international reference and at the same time simple to understand, to reproduce, and to use is needed [9]. However, data suggests that reference curves from other populations may not be representative, thus it is important to develop region-and population-specific reference curves [10–16]. Consequently, gender-specific population-based reference curves are expected to improve the clinical assessment of growth in newborns and evaluation of interventions [17]. In addition, update of the reference curves every 10–15 year is necessary to adjust the curves for changes in the population over time [18–23]. Hence, fetal growth may be assessed in longitudinal studies, clinically or through ultrasound scans. Nevertheless, birth weight and estimated intrauterine fetal weight are not always comparable especially at earlier periods of gestation. Thus, the birth weight data should not be used to calculate intrauterine growth rate [24].

Today clinicians in most developing countries are using the Lubchenco’s reference curve for newborns [1, 25]. However, most neonatology centers in developed countries in Europe use the Niklasson’s curve [19]. Indonesian clinicians, on the other hand, have emphasized the importance of establishing national reference curves. Alisyahbana’s study developed reference curves for 5844 newborns with 34–44 weeks based on data from 14 teaching hospitals in Indonesia from July 1,1990 to June 30,1991 [26]. The result showed that the mean birth weight of Lubchenco’s newborns was significantly different than that from Alisyahbana’s, therefore the Lubchenco’s curve cannot be used as reference curve for Indonesian newborns.

In 1992 the Maternal-Perinatal (MP) team was established in Yogyakarta with the aim of conducting MP audits and creating an MP database in the district hospitals including data collection on birth weight, supine length and head circumference of newborns. The aims of this study were to update the reference curves for birth weight, supine length and head circumference for Yogyakarta, Indonesia and to compare birth weight curves of boys and girls, first child and later children, and the ones in the previous studies.

## Methods

### Study population and study period

The study was conducted in Yogyakarta Special Territory (YST) whose population is made up of various ethnics in Indonesia. Nevertheless it has not represented the population of Indonesia as a whole. YST consist of five districts. Each district is served by a district hospital and a couple of health centers, of which only one was equipped for deliveries, and the referral hospital Sardjito. During the study period January 1, 1998 to December 31, 2007 all deliveries at Sardjito Hospital, the five district hospitals, and the five health centers equipped for deliveries were recorded. Approximately, 80% of the newborns in YST were delivered by trained health personnel, 65% of whom were delivered in Sardjito Hospital, five district hospitals and five health centers; the remaining 35% was delivered in private hospitals, maternity clinics, midwife clinics or at home by midwives [27].

Our study population consisted of all newborns delivered at Sardjito Hospital, five district hospitals, five health centers and those referred from other health facilities within 24 h of birth.

Lubchenco [1, 25], Niklasson [19], and Alisyahbana [26] presented birth weight using gestational age curves for singleton, live born, and healthy newborns. The study population of Lubchenco was collected from Colorado General Hospital, Niklassons from the Swedish Medical Birth Register and it covers the whole Sweden, and Alisyahbana from 14 teaching hospitals in Indonesia (Table 1).

**Table 1:** A comparison of the present study with the previous studies

Reference	Study area	Study population	Study period	Sample size	Subjects				Analysis		
					All/live births	All/Singleton	GA (weeks) Method	Congenital anomalies included	Gender	Mean ± SD by GA	Percentiles by GA
Lubchenco [1, 25]	US (Denver, Colorado)	Colorado General Hospital	1948–61	7827	Live	All	24–42 LMP	No	Yes	No	Yes
Niklasson [19]	Sweden	Medical birth registration	1977–81	475,588	Live	Singleton	28–42 LMP & USG	No	Yes	Yes	No
Kramer [18]	Canada, except Toronto	Provinces	1994–96	676,605	All	Singleton	22–43 USG	Yes	Yes	Yes	Yes
Alisyahbana [26]	Indonesia	14 teaching hospitals	1990–91	5844	Live	Singleton	34-44 LMP	No	Yes	No	Yes
Ulrich M [12]	Denmark (Odense)	Residents	1978	906	Live	Singleton	25–43 USG & Dubowitz	No	Yes	Yes	No
Matthai [24]	India (Velore)	Christian hospital (*n* = 13,217)	1991–94	11,641	Live	Singleton	37–41 Clinical &USG	No (normal)	Yes	No	Yes (only 10, 50, 90)
Fok [20]	Hongkong	Chinese origin (*n* = 104,258)	1998–2001	10,339	Live	Singleton	24–43 (USG & Ballard)	No	Yes	Yes	Yes
Visser [21]	The Netherland	The Netherlands Perinatal Registry (*n* = 183,000)	2001	176,000	Live & intrapartum death	Singleton	25 onwards LMP &USG	Yes	Yes	Yes	Yes
Present study	Indonesia (Yogyakarta)	Sardjito, 5 district hospitals, & 5 health centers (*n* = 59,609)	1998–2007	54,599	Live	Singleton	26–42 (Dubowitz)	No	Yes	Yes	Yes

### Maternal-Perinatal database

The study was conducted by MP team based on MP database. The MP database in the district hospitals is part of MP audit, which is a district-based audit of maternal and perinatal mortality. The MP audit was introduced in Indonesia as a tool for continuous surveillance of the maternal-perinatal mortality and quality assurance of the obstetric and perinatal services into the domain of district health system [28, 29].

The MP database was run in every district hospital by filling in the MP form daily. The data were validated monthly by the local team before they were sent to the MP center at the beginning of the next month and were computerized by a trained secretary. The data generation process from data collection, field editing, data form submission to the data center, and to data entry were continuously monitored to identify errors and logical inconsistencies.

In Indonesia, primary health care services are conducted in health centers. The district hospitals are secondary health facilities that provide referral services in that area. Tertiary health facilities are made available at teaching hospitals, which are usually found in the capital of a province. However, for provinces without a teaching hospital, the services are provided by the provincial hospital, a government hospital in the capital of the province.

The forms from the five district hospitals in YST were submitted to the MP center at Sardjito Hospital until 2001, meanwhile the MP team in the center checked and entered the data. However, from 2002 onwards all facilities were checked and they entered the data by themselves. Therefore the 1998–2001 data were available in the MP center while the 2002–2007 data were available in the health facilities. Unfortunately, an earth-quake struck the area in May 2006 and damaged the soft copy in computers, thus causing most of the data to be re-entered from the MP forms.

The MP database contained information from the mother’s delivery to the neonatal period for each individual in the maternity and newborns facilities in YST. The newborns were followed up until they were discharged from the facilities. Trained health personnel filled in the MP forms. They contained information on identity, characteristics of the mothers, their pregnancy and delivery, and the newborns.

### Inclusion and exclusion criteria

Only live singletons with recorded gestational ages between 26 to 42 weeks and the exact time of admission to the neonatal facility were included in the study; meanwhile those with severely ill conditions (severe asphyxia, severe cardio-respiratory distress, etc.), major congenital anomaly, and those admitted >24 h of age were excluded.

### Assessment of gestational age

In most developing countries, women especially in rural areas are unaware of the exact date of their last menstrual period (LMP). Thus, they could not calculate the expected date of delivery using the first date of the last menstrual period. Dubowitz [30] developed a clinical assessment of gestational age for newborns. A scoring system for gestational age, based on 10 neurologic and 11 external criteria. The correlation coefficient for the total score against gestation was 0.93. The error of prediction of a single score was 1.02 weeks and of the average of two independent assessments was 0.7 weeks. The method gives consistent results within the first 5 days and is equally reliable in the first 24 h of life. The scoring system is more objective and reproducible than trying to guess the gestational age on the presence or absence of individual signs. In the study, gestational age was based on clinical assessment of gestational age according to Dubowitz score and was verified by the mother’s last normal menstrual period in completed weeks.

### Measurements

Birth weight, supine length, and head circumference were measured immediately after delivery. All infants were weighed to the nearest 10 g on a balance scale (readjusted using standardized weight as part of routine care). The length was measured using a measuring board with supports for the head and feet to the nearest cm. The head circumference was recorded using a measuring tape to the nearest cm. Training and standardization in anthropometric measurements of weight, length, head circumference, and clinical assessment of gestational age by Dubowitz score were carried out in December 1997. All measurements were examined by trained nurses.

### Data analysis

Data analysis was performed using SPSS version 19. Tables and graphs presented means and standard deviations (SDs) and the 3^th^, 5^th^, 10^th^, 25^th^, 50^th^ (median), 75^th^, 90^th^, 95^th^, 97^th^ percentiles by gestational age relevant for clinicians in classifying newborns under their care and to researchers as well as public policy makers in comparison to geographic differences and temporal trends in birth weight for gestational ages in population. All analyses were performed separately for boys and girls. Distribution of birth weight, supine length, head circumference at the corrected gestational ages was smoothened by a third degree polynomial function. Curves were produced using Microsoft Excel 2010.

Difference in mean birth weight between boys and girls, as well as first and later-born for each gestational age was analyzed using Student’s *t*-test. In the birth order of children, the term “first” refers to the 1^st^ child, and “later” refers to second child and so on. The weight-length ratio was calculated according to Rohrer’s Ponderal index (PI); 100 x weight in grams/length [3] in centimeters and was classified by tertiles into 3 groups; low, average, or high [31]. The PI was then calculated and classified into low, average and high.

## Results

From January 1998 to December 2007 there were 59,609 births. Most of the infants (83.2%) were born in Sardjito Hospital, five district hospitals, and five health centers, whereas the others (16.8%) were born in other hospitals, health centers, midwife clinics, at home, and were admitted to the study setting before 24 h. In this study there were 54,599 subjects in total. Mean birth weight was 2,964 g and there was no difference in birth weight over time.

**Fig. 1:**
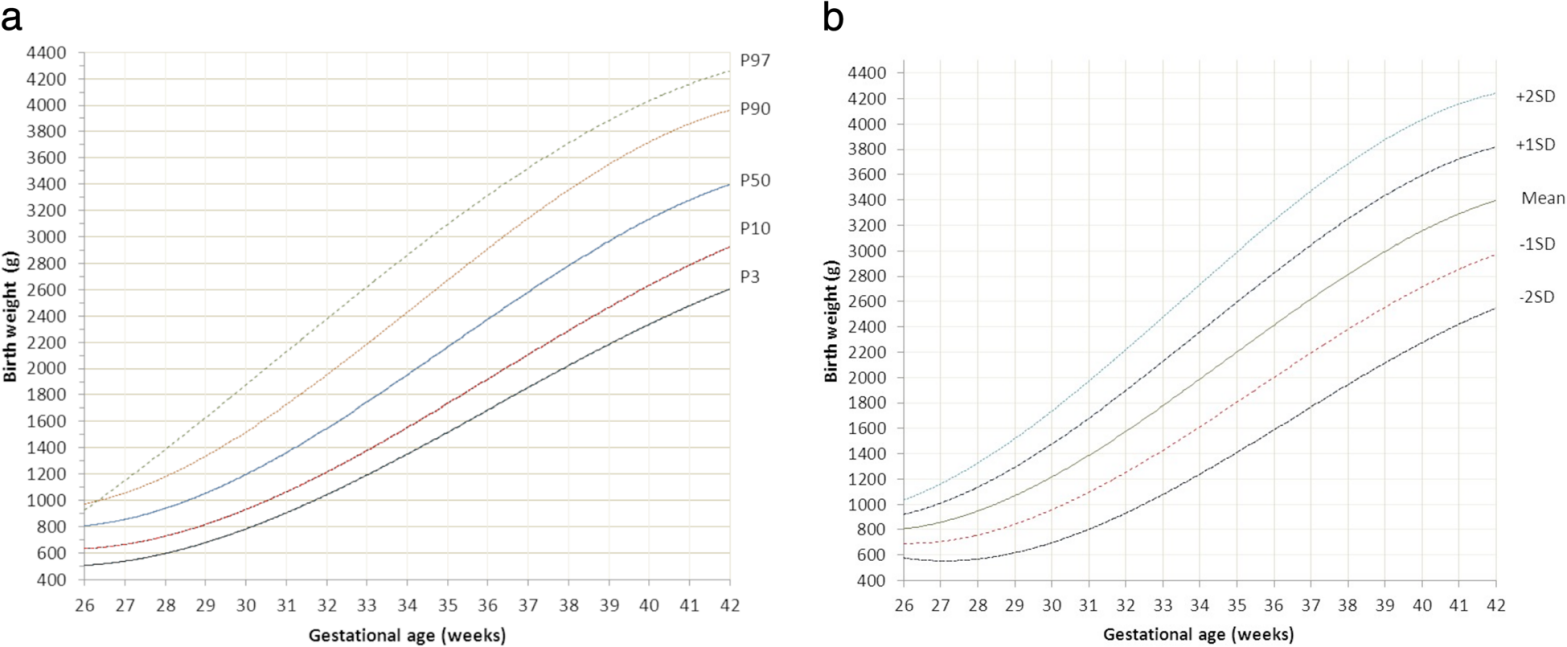
**a** Smoothened percentiles for boys’ birth weight by gestational age. **b**. Smoothened mean and standard deviations for boys’ birth weight by gestational age

**Fig. 2:**
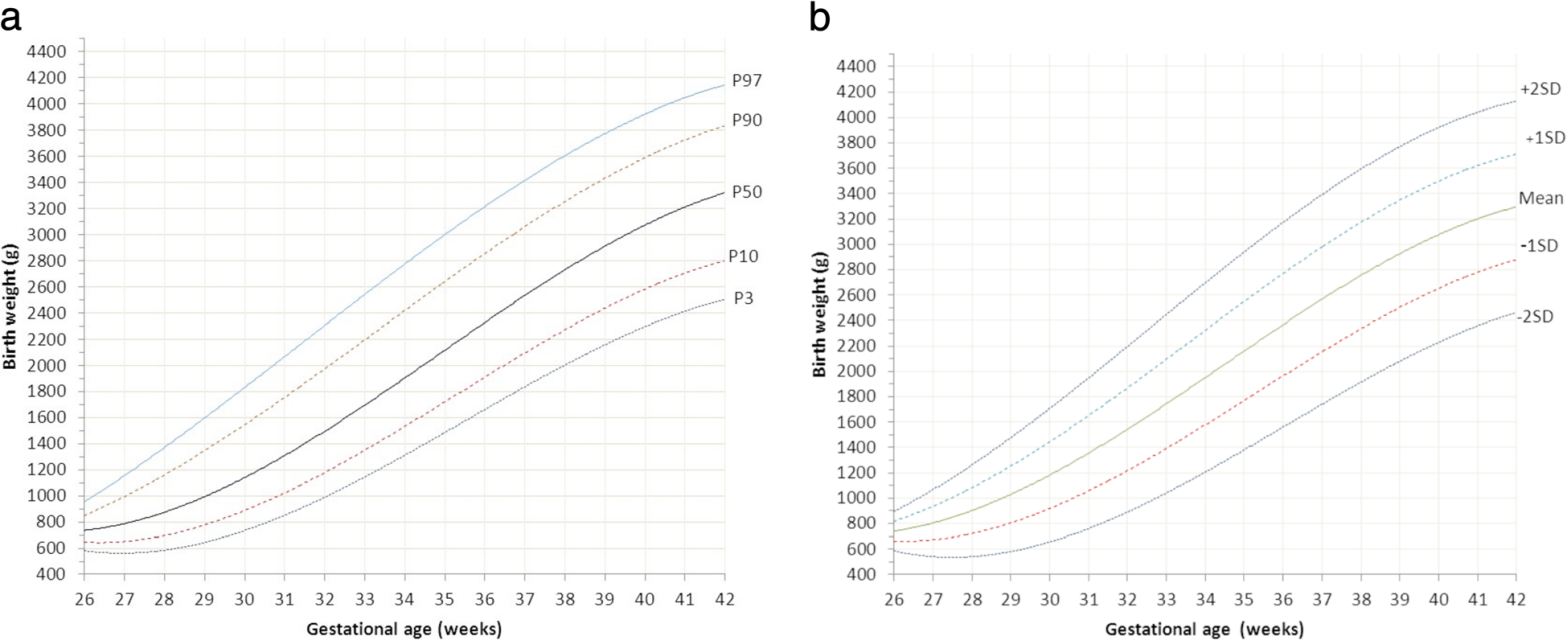
**a** Smoothened percentiles for girls’ birth weight by gestational age. **b**. Smoothened mean and standard deviations for girls’ birth weight by gestational age

**Fig. 3:**
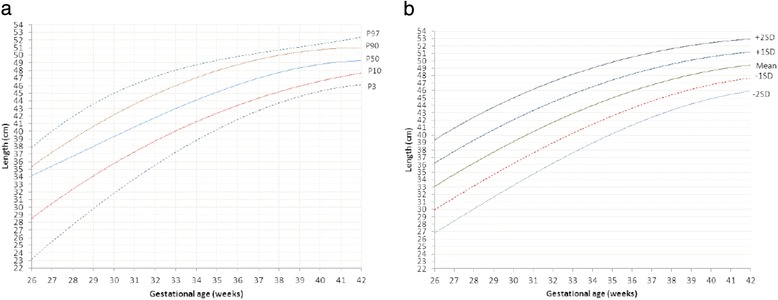
**a** Smoothened percentiles for boys’ length by gestational age. **b**. Smoothened mean and standard deviations for boys’ length by gestational age

**Fig. 4:**
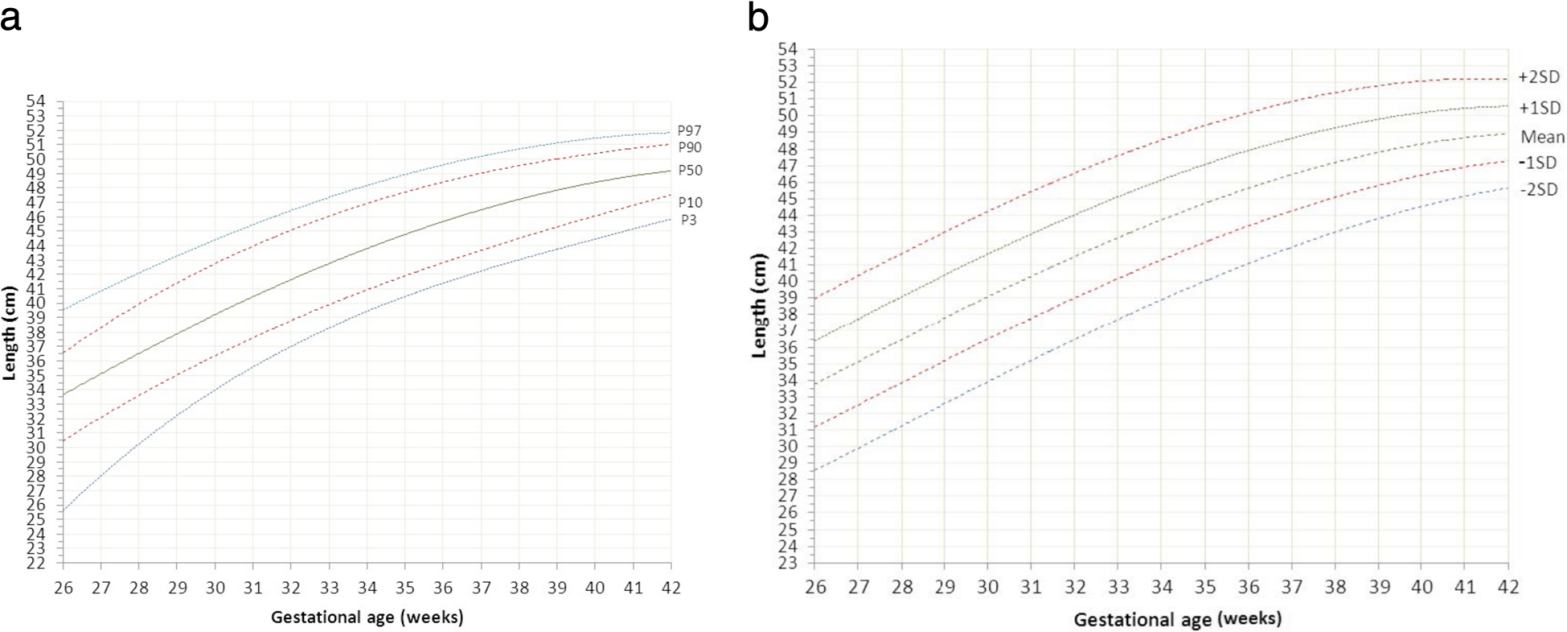
**a** Smoothened percentiles for girls’ length by gestational age. **b**. Smoothened mean and standard deviations for girls’ length by gestational age

**Fig. 5:**
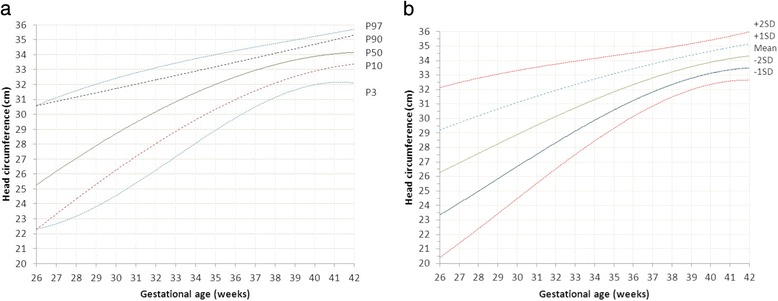
**a** Smoothened percentiles for boys’ head circumference by gestational age. **b**. Smoothened mean standard deviations for boys’ head circumference by gestational age

**Fig. 6:**
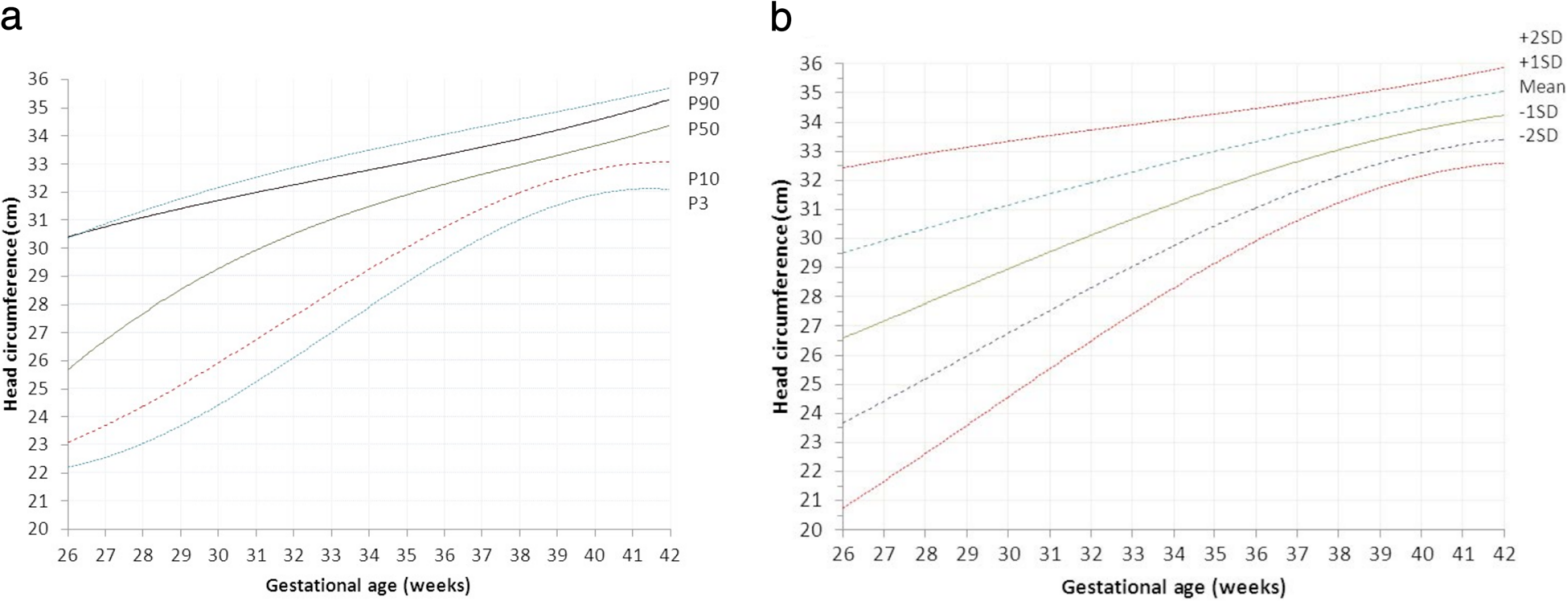
**a** Smoothened percentiles for girls’ head circumference by gestational age. **b**. Smoothened mean standard deviations for girls’ head circumference by gestational age

Sardjito Hospital, the five district hospitals, and the five health centers in YST contributed with 25%, 56% and 19% of the newborns respectively. First child constituted 26,189 (48.0%) and later child was 28,410 (52.0%). The numbers of eligible infants for birth weight, length and head circumference were 54,599, 52,261 and 48,109 respectively (53.3% boys and 46.7% girls) (Table 2). Mean ± SD, percentiles 3, 5, 10, 25, 50, 75, 90, 95, 97 of birth weight, length, and head circumferences for boys and girls were presented in Tables 3, 4, 5. Smoothed curves of birth weight, length, and head circumference for boys and girls were presented in Figs. 1, 2, 3, 4, 5, 6.

**Table 2:** Basic characteristics of the study population (*n* = 54,599)

Characteristic	Category	No	%
Health facility	Sardjito hospital	13,726	25.1
	District hospitals	30,574	56.0
	Health centers	10,299	18.9
Gender	Boys	29,112	53.3
	Girls	25,487	46.7
Birth order	First (1^st^ child)	26,189	48.0
	Later (≥2^nd^ child)	28,410	52.0
Admitted to neonatal ward	Born in the hospital/health centre	45,414	83.2
	Referred <24 h	9,185	16.8
Education of mother (years)	≤5	1,803	3.8
	6–12	40,196	82.7
	≥13	6,576	13.5
Age of mother (years)	≤19	1,770	3.3
	20–34	43,737	81.0
	≥35	8,456	15.7
Number of registered infants	Birth weight	54,599	100
	Length	52,261	95.7
	Head circumference	48,109	88.1

**Table 3:** Birth weight for boys and girls by gestational age in weeks

GA (w)	No of cases	Mean (g)	SD	Birth weight Percentiles (g)								
				P3	P5	P10	P25	P50	P75	P90	P95	P97
*Boys*												
26	55	768.1	170.2	500	500	506	600	750	900	1000	1060	1103
27	39	866.6	152.8	520	600	700	750	850	1000	1100	1100	1100
28	50	968.7	152.9	600	600	800	900	1000	1050	1100	1168	1289
29	52	1057	157.0	600	750	900	1000	1085	1130	1235	1331	1412
30	70	1246	202.3	820	950	1000	1100	1205	1400	1547	1623	1667
31	89	1409	282.3	1050	1063	1100	1200	1380	1525	1700	2025	2318
32	223	1705	377.4	1172	1200	1300	1450	1650	1900	2192	2500	2600
33	258	1750	442.7	1200	1200	1250	1400	1700	2000	2219	2562	2837
34	473	1917	407.1	1200	1350	1400	1650	1900	2200	2400	2600	2939
35	541	2035	378.5	1350	1400	1552	1800	2000	2250	2400	2595	2787
36	868	2382	430.7	1650	1750	1900	2100	2350	2550	3000	3216	3400
37	1576	2643	427.1	1800	1999	2150	2450	2600	2900	3200	3400	3500
38	3799	2862	404.8	2100	2200	2400	2600	2800	3100	3400	3550	3700
39	6915	3069	382.3	2310	2496	2600	2850	3050	3300	3500	3700	3800
40	8755	3184	410.5	2414	2540	2700	2950	3180	3400	3700	3900	4000
41	3812	3358	445.0	2500	2600	2800	3100	3400	3650	3900	4000	4200
42	1537	3295	463.5	2500	2600	2800	3000	3250	3500	3950	4182	4300
*Girls*												
26	48	680.8	134.8	500	500	500	600	650	767	900	967	991
27	41	844.3	156.2	600	609	700	770	800	900	1040	1100	1396
28	59	945.3	119.2	600	700	800	900	1000	1000	1100	1100	1166
29	42	1023	109.6	765	800	900	994	1000	1100	1141	1193	1271
30	49	1151	230.2	675	760	850	1000	1100	1300	1500	1575	1665
31	74	1374	294.1	825	975	1100	1200	1340	1500	1725	2050	2200
32	171	1711	441.3	1100	1150	1200	1400	1600	1900	2480	2608	2700
33	211	1692	406.2	1200	1200	1250	1400	1600	1850	2200	2520	2800
34	392	1862	386.5	1200	1250	1400	1568	1875	2100	2300	2400	2500
35	515	2046	386.3	1400	1500	1600	1800	2000	2250	2400	2600	2890
36	812	2335	436.8	1500	1700	1823	2100	2300	2500	2900	3200	3300
37	1384	2589	397.0	1800	1925	2145	2400	2500	2800	3100	3300	3400
38	3318	2800	375.1	2100	2200	2400	2600	2800	3000	3250	3450	3600
39	6065	2997	371.3	2300	2400	2600	2750	3000	3200	3450	3600	3700
40	7607	3099	393.6	2400	2500	2600	2850	3100	3350	3560	3750	3900
41	3254	3259	447.4	2400	2500	2700	3000	3300	3550	3800	4000	4050
42	1445	3208	447.3	2400	2500	2700	2900	3200	3500	3800	4000	4200

*GA* Gestational Age; *SD* Standard Deviation; *P* Percentiles; *g* gram; *w* week

**Table 4:** Length supine of boys and girls by gestational age in weeks

GA (w)	No of cases	Mean (cm)	SD	Lenght Supine Percentiles (cm)								
				P3	P5	P10	P25	P50	P75	P90	P95	P97
*Boys*												
26	54	33.6	2.73	25	28	31	32	34	35	36	36	37
27	37	33.9	3.88	24	24	25	33	35	36	37	37	40
28	50	35.9	2.94	25	30	35	35	36	37	38	40	43
29	50	37.7	3.18	29	35	35	36	38	39	40	43	45
30	67	39.4	3.01	31	35	36	37	40	41	43	44	44
31	89	41.3	2.02	37	37	39	40	41	42	44	45	45
32	223	42.6	2.27	40	40	40	41	43	44	45	47	47
33	258	42.1	2.89	36	37	38	41	42	44	46	47	48
34	413	43.4	3.08	37	38	40	42	44	46	47	48	49
35	475	44.0	3.19	38	38	40	42	44	46	48	48	49
36	868	45.9	2.01	42	43	44	45	46	47	49	49	50
37	1470	47.0	2.04	43	43	45	46	47	48	49	50	50
38	3778	47.8	1.86	44	45	46	47	48	49	50	50	51
39	6754	48.4	1.74	45	46	47	48	49	50	50	51	51
40	8168	48.8	1.80	45	46	47	48	49	50	51	51	52
41	3584	49.1	2.04	46	46	47	48	49	50	51	52	52
42	1527	49.1	1.76	46	46	47	48	49	50	51	52	52
*Girls*												
26	43	34.1	2.91	26	28	30	33	34	36	37	39	40
27	37	34.8	2.51	25	31	32	34	35	36	38	39	40
28	59	35.9	2.07	33	33	34	35	36	37	40	40	42
29	41	37.7	2.84	30	31	35	36	37	40	42	43	43
30	49	38.8	2.86	34	34	35	36	40	41	42	43	44
31	74	41.3	2.08	38	38	39	40	41	42	45	45	47
32	171	42.9	2.16	40	40	41	41	43	44	46	47	47
33	210	41.9	2.45	37	38	39	40	42	43	45	46	47
34	351	43.1	3.25	37	37	39	41	43	45	47	48	48
35	457	44.0	2.85	38	39	41	42	44	46	48	48	49
36	812	45.7	2.20	41	42	43	45	46	47	48	49	50
37	1304	46.7	1.96	43	43	44	46	47	48	49	50	50
38	3299	47.4	1.78	44	45	45	46	47	49	50	50	51
39	5933	48.0	1.70	45	45	46	47	48	49	50	50	51
40	7074	48.4	1.79	45	46	46	47	48	49	50	51	51
41	3043	48.7	2.04	45	46	47	48	49	50	51	51	52
42	1439	48.8	1.70	45	46	47	48	49	50	51	52	52

*GA* Gestational Age; *SD* Standard Deviation; *P* Percentiles; *cm* centimeter; *w* week

**Table 5:** Head circumference of boys’ and girls’ by gestational age in weeks

GA (w)	No of cases	Mean (cm)	SD	Head Circumferences Percentiles (cm)								
				P3	P5	P10	P25	P50	P75	P90	P95	P97
*Boys*												
26	50	26.7	2.79	22	22	23	24	26	30	30	30	30
27	33	25.9	2.48	23	23	23	24	25	28	30	31	31
28	42	27.8	3.19	23	23	24	25	27	30	33	33	33
29	35	29.0	2.83	24	25	26	27	28	32	33	33	33
30	63	28.6	1.89	25	25	26	27	29	30	31	31	31
31	89	29.2	1.80	25	26	27	28	29	31	31	32	32
32	223	31.3	1.40	27	28	30	31	32	32	32	33	33
33	256	30.4	1.86	26	27	28	30	31	32	32	33	34
34	398	31.0	1.42	28	29	29	30	31	32	33	34	34
35	465	31.2	1.19	29	29	30	31	31	32	33	33	34
36	868	32.6	1.09	30	31	32	32	33	34	34	34	34
37	669	32.7	1.18	30	30	31	32	33	34	34	34	35
38	3534	33.3	0.871	32	32	32	33	34	34	34	35	35
39	6296	33.7	0.778	32	32	33	34	34	34	35	35	35
40	7871	33.9	0.751	32	32	33	34	34	34	35	35	35
41	3463	34.2	0.763	32	33	34	34	34	35	35	36	36
42	1289	34.1	0.809	32	33	33	34	34	35	35	36	36
*Girls*												
26	36	26.6	2.81	22	22	23	24	26	30	30	30	30
27	31	27.0	2.53	23	24	24	25	26	30	30	30	30
28	46	27.4	3.16	22	23	24	25	27	30	32	33	33
29	31	29.5	2.36	25	26	26	28	30	31	33	33	33
30	41	28.4	2.30	23	23	24	27	29	30	31	31	31
31	74	29.3	1.75	25	26	27	28	30	31	31	32	32
32	171	31.1	1.53	27	28	29	30	32	32	33	33	33
33	207	30.3	1.75	27	27	28	29	30	32	32	33	33
34	342	30.8	1.32	28	28	29	30	31	32	32	33	33
35	452	31.2	1.32	28	29	30	31	31	32	33	33	34
36	812	32.4	1.23	30	30	31	32	32	33	34	34	34
37	608	32.7	1.26	30	30	31	32	33	34	34	34	35
38	3088	33.2	0.848	31	32	32	33	34	34	34	34	35
39	5544	33.6	0.774	32	32	33	34	34	34	35	35	35
40	6817	33.8	0.752	32	32	33	34	34	34	35	35	35
41	2964	34.1	0.778	32	33	33	34	34	35	35	35	36
42	1201	34.0	0.835	32	32	33	34	34	35	35	35	36

*GA* Gestational Age; *SD* Standard Deviation; *P* Percentiles; *cm* centimeter; *w* week

**Fig. 7:**
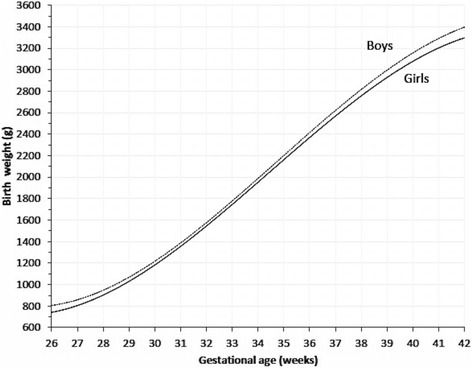
Mean birth weight for boys’ and girls’ by gestational age

**Fig. 8:**
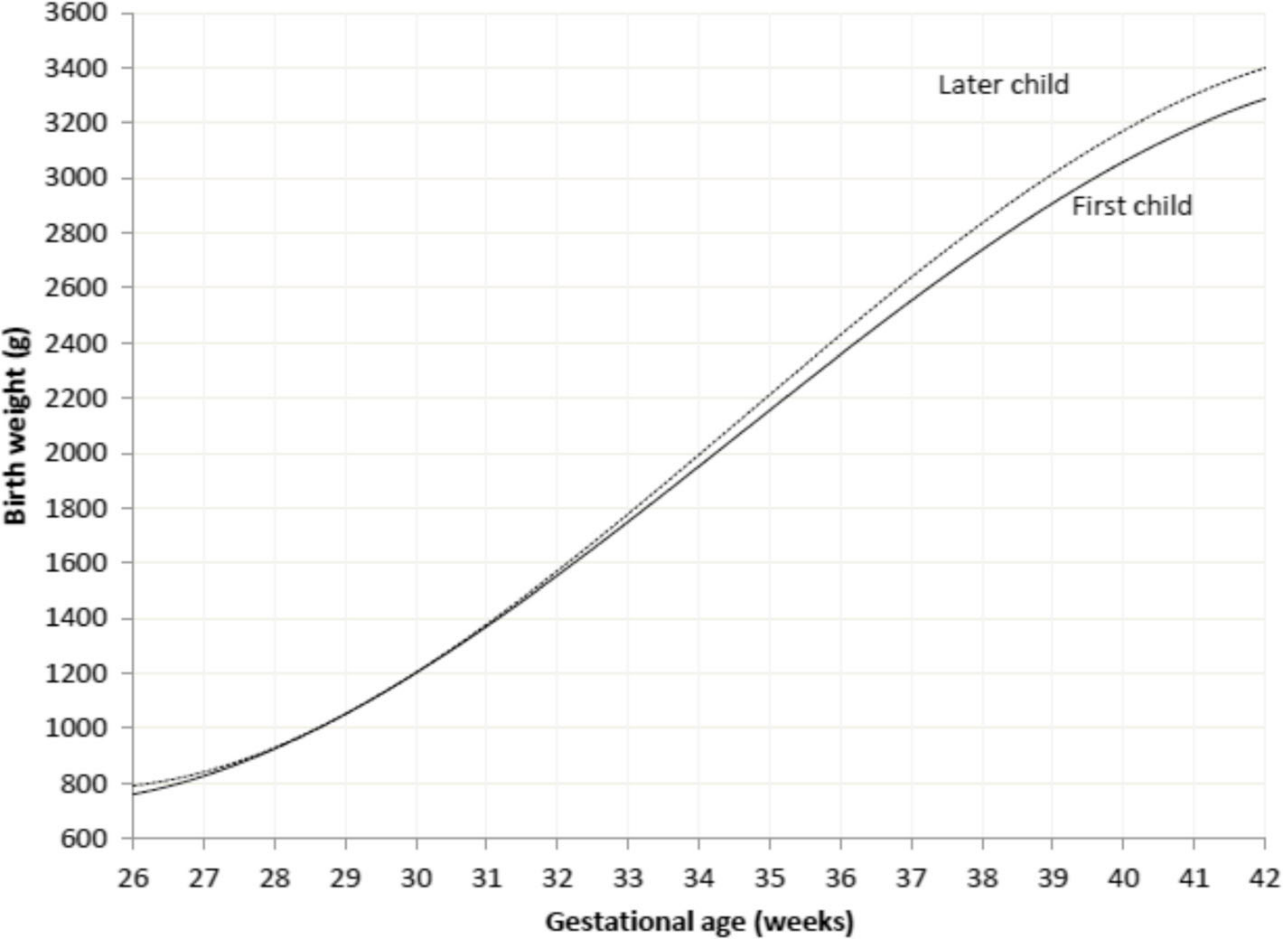
Mean birth weight for 1st and ≥2nd child by gestational age

At term (37–42 weeks gestational age) mean birth weight for each gestational age was significantly higher for boys than for girls (Table 6, Fig. 7) and for later born than for first born (Table 7, Fig. 8).

**Table 6:** Mean birth weight, standard deviation, ponderal index, classification for boys and girls by gestational age

GA (w)	Boys			Girls			*p*	Boys		Girls	
	No of cases	Mean (g)	SD	No of cases	Mean (g)	SD		PI	C	PI	C
26	55	768.1	170.2	48	680.8	134.8	0.005	2.1	L	1.7	L
27	39	866.6	152.8	41	844.3	156.2	0.52	2.4	L	2.0	L
28	50	968.7	152.9	59	945.3	119.2	0.37	2.2	L	2.1	L
29	52	1057	157.0	42	1023	109.6	0.25	2.0	L	1.9	L
30	70	1246	202.3	49	1151	230.2	0.019	2.1	L	2.0	L
31	89	1409	282.3	74	1374	294.1	0.45	2.0	L	1.9	L
32	223	1705	377.5	171	1711	441.3	0.87	2.2	L	2.2	L
33	258	1750	442.7	211	1692	406.2	0.15	2.3	L	2.3	L
34	473	1917	407.1	392	1862	386.5	0.043	2.4	L	2.4	L
35	541	2035	378.5	515	2046	386.3	0.64	2.4	L	2.4	L
36	868	2382	430.7	812	2335	436.8	0.026	2.4	L	2.4	L
37	1576	2643	427.1	1384	2589	397.0	<0.001	2.5	A	2.5	A
38	3799	2862	404.8	3318	2800	375.1	<0.001	2.6	A	2.6	A
39	6915	3069	382.3	6065	2997	371.4	<0.001	2.7	A	2.7	A
40	8755	3184	410.5	7607	3099	393.6	<0.001	2.8	A	2.7	A
41	3812	3358	445.0	3254	3259	447.4	<0.001	2.8	A	2.8	A
42	1537	3295	463.5	1445	3208	447.3	<0.001	2.8	A	2.8	A

*C* Classification; *L* Low, *A* Average, *H* High; *GA* Gestational Age; *SD* Standard Deviation; *P* Percentiles; *g* gram; *w* week

**Table 7:** Mean birth weight, standard deviation, Ponderal index and classification by birth order and gestational age

GA (w)	First child			Later children			*p*	First child		Later children	
	No of cases	Mean (g)	SD	No of cases	Mean (g)	SD		PI	C	PI	C
26	48	723.6	173.7	55	730.7	148.8	0.83	2.0	L	1.9	L
27	40	832.8	145.3	40	877.5	160.8	0.18	2.2	L	2.3	L
28	60	951.6	139.1	49	961.3	132.2	0.71	2.1	L	2.1	L
29	56	1041	107.7	38	1043	175.3	0.94	2.0	L	2.0	L
30	57	1199	203.6	62	1214	232.6	0.70	2.1	L	2.0	L
31	84	1413	315.6	79	1372	254.2	0.37	2.0	L	2.0	L
32	214	1698	393.4	180	1720	421.0	0.58	2.2	L	2.2	L
33	228	1689	407.1	241	1757	443.7	0.083	2.3	L	2.3	L
34	508	1874	386.2	357	1917	414.8	0.12	2.3	L	2.4	L
35	628	2034	361.9	428	2049	410.4	0.54	2.4	L	2.4	L
36	906	2328	390.9	774	2396	477.6	0.002	2.4	L	2.5	A
37	1525	2569	381.3	1435	2669	440.7	<0.001	2.5	A	2.6	A
38	3510	2783	361.3	3607	2883	414.7	<0.001	2.6	A	2.7	A
39	6159	2983	359.5	6821	3083	389.7	<0.001	2.7	A	2.7	A
40	7527	3075	377.2	8835	3204	418.0	<0.001	2.7	A	2.8	A
41	3289	3246	443.1	3777	3370	445.8	<0.001	2.8	A	2.9	H
42	1350	3199	440.7	1632	3297	466.9	<0.001	2.7	A	2.8	A

*C* Classification; *L* Low; *A* Average; *H* High; *GA* Gestational Age; *SD* Standard Deviation; *P* Percentiles; *g* gram; w week

**Fig. 9:**
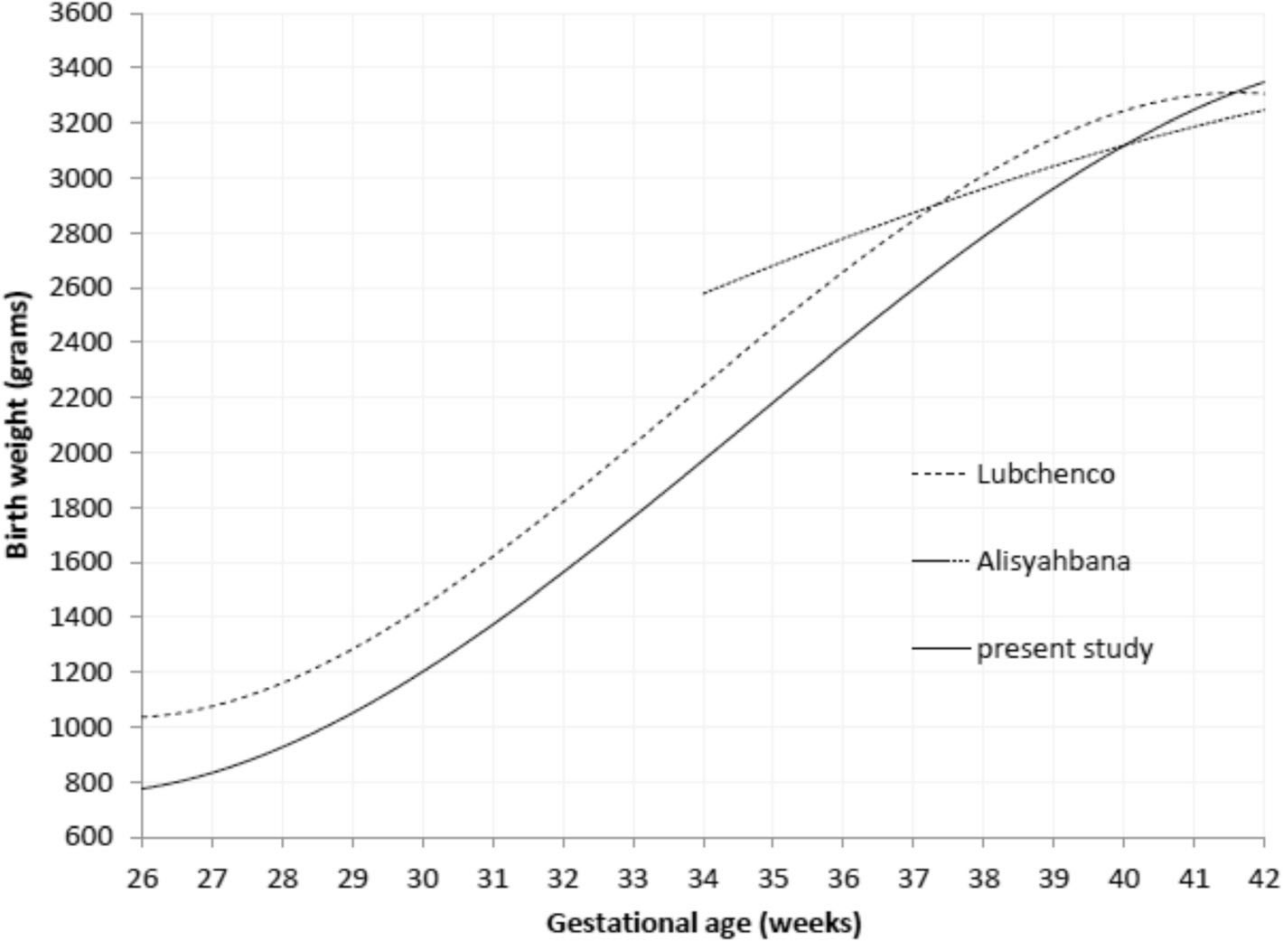
Mean birth weight by gestational age according to Lubchenco’s, Alisyahbana’s and present study

For gestational age ≥39 weeks there was a striking similarity in mean birth weight among Lubchenco’s, Alisyahbana’s, and our study. The mean birth weight for gestational age ≤38 weeks was lower in our study than that in Lubchenco’s. Gestational age 34–37 weeks presented the highest mean birth weight in Alisyahbana’s but the lowest in our study (Table 8, Fig. 9).

**Table 8:** Mean birth weight, Ponderal index, classification in Lubchenco’s, Alisyahbana’s and present study by gestational age

GA (w)	Lubchenco				Alisyahbana				Present study			
	No of cases	BW (g)	PI	C	No of cases	BW (g)	PI	C	No of cases	BW (g)	PI	C
26	68	1001	2.2	L					103	727	1.9	L
27	72	1065	2.2	L					80	855	2.1	L
28	118	1236	2.2	L					109	956	2.1	L
29	143	1300	2.3	L					94	1042	2.0	L
30	109	1484	2.3	L					119	1207	2.0	L
31	147	1590	2.4	L					163	1393	1.9	L
32	124	1732	2.4	L					394	1708	2.2	L
33	118	1957	2.4	L					469	1724	2.3	L
34	145	2278	2.5	A	43	2553	2.5	A	865	1892	2.3	L
35	188	2483	2.5	A	70	2704	2.6	A	1056	2040	2.4	L
36	202	2753	2.5	A	136	2849	2.4	L	1680	2359	2.5	A
37	372	2800	2.6	A	262	2819	2.5	A	2960	2618	2.5	A
38	636	3025	2.6	A	565	2903	2.5	A	7117	2833	2.6	A
39	1010	3130	2.6	A	1309	3066	2.6	A	12980	3035	2.7	A
40	1164	3226	2.6	A	1710	3146	2.5	A	16362	3145	2.7	A
41	632	3307	2.6	A	962	3205	2.6	A	7066	3312	2.8	A
42	336	3308	2.6	A	446	3228	2.6	A	2982	3253	2.7	A
Total	5584				5503				54599			

*C* Classification; *L* Low; *A* Average; *H* High; *GA* Gestational Age; *PI* Ponderal Index; *BW* Birth Weight; g gram; w week

Tertiles of PI of our study were low (<2.5), average (2.5–2.8) and high (>2.8). The PI of term boys, girls, first and later children in our study were classified into average group. In the preterm, however, it was classified into low group (Tables 6 and 7). The PI for gestational age was consequently lower in our study than in Lubchenco’s. The gestational age ≥39 weeks was higher in our study than it was in Lubchenco’s and Alisyahbana’s (Table 8).

## Discussion

Our study presented girls and boys for birth weight, length and head circumference based on the local data. One of the weaknesses of our study was that it did not have enough low-gestational age infants. Therefore the application of the curve in low gestational age infant must be done carefully.

Moreover, comparison of each gestational age showed higher significance in at term only, but not in preterm. The result was similar to the study by Fok [20] whereby the mean birth weight of boys consistently exceeded that of girls at 36 weeks or more gestational ages. Lubchenco [1] showed differences of approximately 100 g, significant between boys and girls at 38 to 41 weeks. Skjaerven [16] explained that the effects at 40 weeks in boys were heavier than those in girls. However, Olsen [32] found that all were statistically different by age group, and most were considered clinically different enough. This illustrates the necessity to create separate charts for boys and girls.

Skjaerven [16] pointed out that later children at 40 weeks were between 130–150 g heavier than first children. This was similar to our study which showed that each gestational age, at term later-born children were significantly 100–130 g heavier (*p* < 0.001) than first-born children. In preterm there was no significant difference, though. Nevertheless, Alisyahbana reported that for every gestational age and percentile, later-born children were heavier than first born-children [27].

We could not compare the mean birth weight for each gestational age in our study and that in the previous studies by Lubchencho and Alisyahbana, since there was no information on standard deviation. Thus, the comparison was based on mean birth weight for sexes combine because no information of separated boys and girls was found in Alisyahbana’s. Similarly, comparison of our study and Lubchenco’s showed that for gestational age ≤38 weeks the mean birth weight was lower in our study. This was probably due to the relatively high number of infants with small for gestational age in our population for term and preterm, which needed further investigation.

Compared with Alisyahbana’s study, for gestational age 34–37 weeks the mean birth weight was lower in our study; which was probably due to the differences of sample. Our study had more data from health centers, district hospitals, and 1 teaching hospital, whereas Alisyahbana’s study collected the data from 14 teaching hospitals with middle and high socio-economic status. In addition, the numbers of samples in our study were much higher with updated reference for 26 to 42 weeks gestational age, meanwhile Alisyahbana’s was only 34–42 weeks. Unfortunately, we could not compare our result with Niklasson’s curve [20], since we were not able to find the data in the Niklasson’s articles.

Tertiles of PI for our study were similar to those of Morris’s [31] report, which showed <2.6 low, 2.6–2.8 average and >2.8 high. The PI of at term of boys, girls, first, and later children in our study was at average. However, in the preterm it was low.

Lubchenco [26] reported that there was an increasing weight-length ratio (PI) as gestation progressed; the babies became heavier for length as they approached near full term. Similar to our study, PI was classified into preterm and average in near term (35–36 weeks) and term (>37 weeks).

Thus, the combination of short and low PI at birth may well provide a useful classification of the anthropometric status of the newborns. Infants who were born short with low PI were at risk of mortality and severe morbidity during infancy [31]. The low PI of Lubchenco’s was for gestational age ≤33 weeks, whereas it was for ≤35 weeks in our study. If we found a short newborns <35 weeks of gestational age, therefore, he/she would be at high risk for morbidity and mortality.

Important cut off points for risk assessment of the 3^rd^ and 97^th^ percentiles, −2 SD or +2 SD were added. We expect that these curves would be useful for the care of Indonesian newborns.

## Conclusions

Our study separated girls and boys for birth weight, length and head circumference based on the local data. At term, mean birth weight of boys was significantly higher than that of girls, mean birth weight of first-born children was significantly lower than that of later born-children; but in preterm, both did not suggest significant difference.

For gestational age ≥39 weeks there was mean birth weight similarity to Lubchenco’s, Alisyahbana’s, and our study. When compared with Lubchenco’s study, the mean birth weight for gestational age ≤38 weeks was lower in our study. However, for 34–37 weeks, the mean birth weight in our study was lower than that in Alisyahbana’s study.

The PI of term for boys and girls and first and later-born children was classified into average, whereas that of preterm was classified into low. The PI for gestational age ≤35 weeks was lower in our study than in Lubchenco’s; however, for gestational age ≥39 weeks it was higher in our study than in Lubchenco’s and Alisyahbana’s.

Updated and improved neonatal reference curves for birth weight, supine length, and head circumference are important to classify high risk newborns in specific area and to recognize those requiring attention with regard to recent condition.

## Abbreviations

A: Average
C: Classification
GA: Gestational age
H: High
HC: Head circumference
L: Low
LMP: Last menstrual period
MP: Maternal-perinatal
PI: Ponderal index
SD: Standard deviation
SGA: Small for gestational age
USG: Ultrasonography
YST: Yogyakarta special territory
